# Life Cycle Assessment Applied to Oral Healthcare: A Scoping Review

**DOI:** 10.3390/healthcare14213600

**Published:** 2026-10-23

**Authors:** Diego Rodrigues Aguilar, Nathalia Sernizon Guimarães, Myrelle Leal Campos Sousa, Daniele Lopes Leal, Andrea Franco Pereira, Mauro Henrique Nogueira Guimarães de Abreu

**Affiliations:** 1 School of Dentistry, Universidade Federal de Minas Gerais, Belo Horizonte 31270-901, Brazil; diego_aguilar17@hotmail.com (D.R.A.); myrellesousa21@gmail.com (M.L.C.S.); 2 Department of Nutrition, Universidade Federal de Minas Gerais, Belo Horizonte 30130-100, Brazil; nasernizon@ufmg.br; 3 Department of Community and Preventive Dentistry, Universidade Federal de Minas Gerais, Belo Horizonte 31270-901, Brazil; dandanileal@yahoo.com.br; 4 School of Architecture, Universidade Federal de Minas Gerais, Belo Horizonte 30130-141, Brazil; andreafranco@arq.ufmg.br

**Keywords:** life cycle assessment, oral health, dentistry, environmental impact, sustainable development, scoping review, dental materials, health services

## Abstract

Context/Objectives: Environmental sustainability has become a growing concern in oral health, as dental care involves materials, devices, energy consumption, water usage, transportation, and complex supply chains, resulting in the generation of various types of waste and emissions. Life Cycle Assessment (LCA) offers a methodological framework for evaluating environmental impacts throughout the life cycle of products, procedures, services, and population-level interventions. This scoping review aimed to map how LCA has been applied to assess environmental impacts related to oral health. Methods: A scoping review was conducted following the Joanna Briggs Institute recommendations and reported according to the PRISMA-ScR checklist. Information about the review was made available through a public Open Science Framework project created on 20 February 2026. Primary studies applying LCA to clinical dental procedures, dental materials and devices, oral health services, or population-level preventive interventions were included. Searches were performed in PubMed, Embase, Web of Science Core Collection, and the Cochrane Library, as well as MEDLINE, LILACS, WPRIM, IBECS, and BINACIS through the Virtual Health Library (BVS) interface. The final search was conducted on 11 February 2026, without language or publication year restrictions. Data were narratively synthesized and organized according to study characteristics, methodological aspects, impact categories, environmental hotspots, and reported limitations. Results: Twenty-one studies published between 2014 and 2026 were included, of which 20 (95.2%) had bibliographic publication years of 2020 or later. The evidence was concentrated in Northern Hemisphere countries. Dental materials and devices were the most frequently investigated subjects, followed by clinical procedures, population-level preventive interventions, and oral health services. Among the included publications, openLCA was reported in 17 (81.0%) and ecoinvent in 20 (95.2%). Climate change, expressed as CO_2_ equivalents, was a frequently reported impact category. Reported environmental hotspots included single-use materials, energy consumption, sterilization and reprocessing, transportation, and material manufacturing. The included studies showed heterogeneity in methodological choices and assessment contexts. Conclusions: This scoping review maps the methodological characteristics, environmental hotspots, and research gaps in the application of LCA to oral healthcare. The heterogeneity of the available evidence limits direct comparisons and does not establish the environmental superiority of specific dental practices. Clinical, institutional, and policy decisions require context-specific assessments, functionally equivalent comparisons, and explicit consideration of uncertainty.

## 1. Introduction

Sustainability has moved to the forefront of the debate on health systems as the delivery of care has come to be recognized for its environmental effects as well. Studies on the sector’s environmental footprint indicate that these impacts are not only limited to direct emissions from services, as they also encompass supply chains, energy consumption, healthcare infrastructure, consumption patterns, and the organizational structures of care systems [1,2]. In the field of oral health, this agenda has been incorporated into institutional documents and recent reviews, highlighting the shared responsibility among professionals, administrators, educators, researchers, manufacturers, distributors, and waste management entities [3,4,5,6]. Thus, although recent progress has expanded the integration of sustainability into dentistry, there is still a need to understand, in a more structured manner, how these impacts are distributed throughout the various stages of care and the clinical and organizational decisions that drive them.

Against this backdrop, Life Cycle Assessment (LCA) offers a particularly relevant methodological approach, as it enables the analysis of environmental impacts associated with products, processes, or services throughout their life cycle stages, from raw material extraction to production, transport, use, reprocessing, and final disposal, a concept also known as “cradle-to-grave” [7,8]. This perspective matters for oral health, as dental care combines materials, equipment, clinical processes, and organizational decisions that are difficult to fully understand by looking at only a single treatment stage [9]. By integrating these dimensions into a unified analytical framework, LCA allows for the evaluation of clinical alternatives, materials, services, and preventive strategies with greater environmental consistency. Importantly, a life cycle approach should be distinguished from the number of environmental impact categories assessed. Studies evaluating climate change as the sole impact category may still apply LCA methodology, whereas emissions calculations that do not consider a life cycle approach should not be classified as LCA. In addition, social life cycle assessment represents a related application of the life cycle framework, addressing social indicators that should not be interpreted as environmental impact outcomes [7,8,9].

The strength of LCA also presents a challenge: its results depend on the choices made to define the environmental problem. Objective, scope, functional unit, system boundaries, inventory, and impact categories are not merely technical steps; they define what is recognized as an impact and how that impact is interpreted. Differences in these methodological choices may limit direct comparisons between studies, particularly when assessments involve different functional units, system boundaries, inventory assumptions, and impact assessment methods. Such heterogeneity does not, by itself, indicate methodological inadequacy, but requires careful consideration when interpreting and transferring environmental estimates across settings [7,8,9]. In the healthcare sector, initiatives such as HealthcareLCA underscore the importance of compiling environmental assessments into accessible databases capable of transforming scattered results into information useful for research, management, and clinical practice [10]. In oral health, such an organization is still needed to understand which dimensions of care have been evaluated via LCA, which remain under-explored, and the extent to which available results can support comparable environmental decisions.

Although sustainability in oral health has gained prominence in the literature, it remains unclear how LCA has been incorporated into dental scientific research, or which subjects, methods, and impact categories have structured this application. This scoping review aims to map how LCA has been used to assess environmental impacts related to clinical procedures, dental materials and devices, oral health services, and population-level preventive interventions. The review also identifies related applications of the life cycle framework, distinguishing social indicators from environmental impact outcomes. By organizing this evidence, the study seeks to outline the current state of the field and identify relevant gaps for future research while recognizing that the available evidence does not, by itself, establish the environmental superiority of specific dental practices or support direct clinical and policy recommendations without context-specific assessments and consideration of uncertainty.

## 2. Materials and Methods

This scoping review was conducted to map the extent and characteristics of scientific literature regarding the application of the life cycle assessment LCA to analyze environmental impacts related to oral health. This design was chosen because it is suitable for investigating emerging fields where available evidence is heterogeneous regarding the objects of analysis, methodological approaches, and application contexts [11,12]. This review followed the Joanna Briggs Institute (JBI) recommendations for scoping reviews [13]. The methodological characteristics of the included studies were descriptively characterized, without conducting a formal critical appraisal or applying a validated quality-assessment instrument. This review was written in accordance with guidelines for Preferred Reporting Items for Systematic Reviews and Meta-Analyses Extension for Scoping Reviews (PRISMA-ScR) [14].

Information about this review was made available in a public Open Science Framework (OSF) project created on 20 February 2026 (https://osf.io/b5wc7/overview (accessed on 5 October 2026)). This repository was not considered evidence of prospective protocol registration. The guiding question—“How has Life Cycle Assessment (LCA) been applied to evaluate environmental impacts related to oral health?”—was formulated based on the Population, Concept, and Context (PCC) framework, where P (Population) = dental procedures, products, and services; C (Concept) = LCA and environmental impacts; and C (Context) = dental environments at multiple levels, including clinical, laboratory, hospital, community, and population settings (Table 1) [13].

Primary studies applying the life cycle approach to evaluate environmental impacts associated with dental procedures, materials, equipment, services, or technologies in dental settings were included. Eligibility required the application of a life cycle perspective rather than a minimum number of environmental impact categories. Studies assessing only climate change or carbon footprint were eligible when they employed LCA methodology, whereas emissions calculations without a life cycle approach were excluded. No restrictions were applied regarding language or year of publication. Narrative literature reviews, systematic reviews, scoping reviews, rapid reviews, living reviews, overviews, meta-analyses, editorials, letters to the editor, commentaries, and studies that did not use LCA or were unrelated to oral health were excluded.

Bibliographic searches were conducted in the PubMed, Embase, Web of Science Core Collection, and the Cochrane Library, as well as MEDLINE, LILACS, WPRIM, IBECS, and BINACIS accessed through the Virtual Health Library (BVS) interface. The final search was conducted on 11 February 2026. Search strategies combined descriptors from controlled vocabularies (MeSH, DeCS, and Emtree) with synonyms and free-text terms related to dentistry and LCA, linked by the Boolean operators AND and OR, and adapted for each database. The search strings are presented in Appendix A (Table A1).

**Figure 1:**
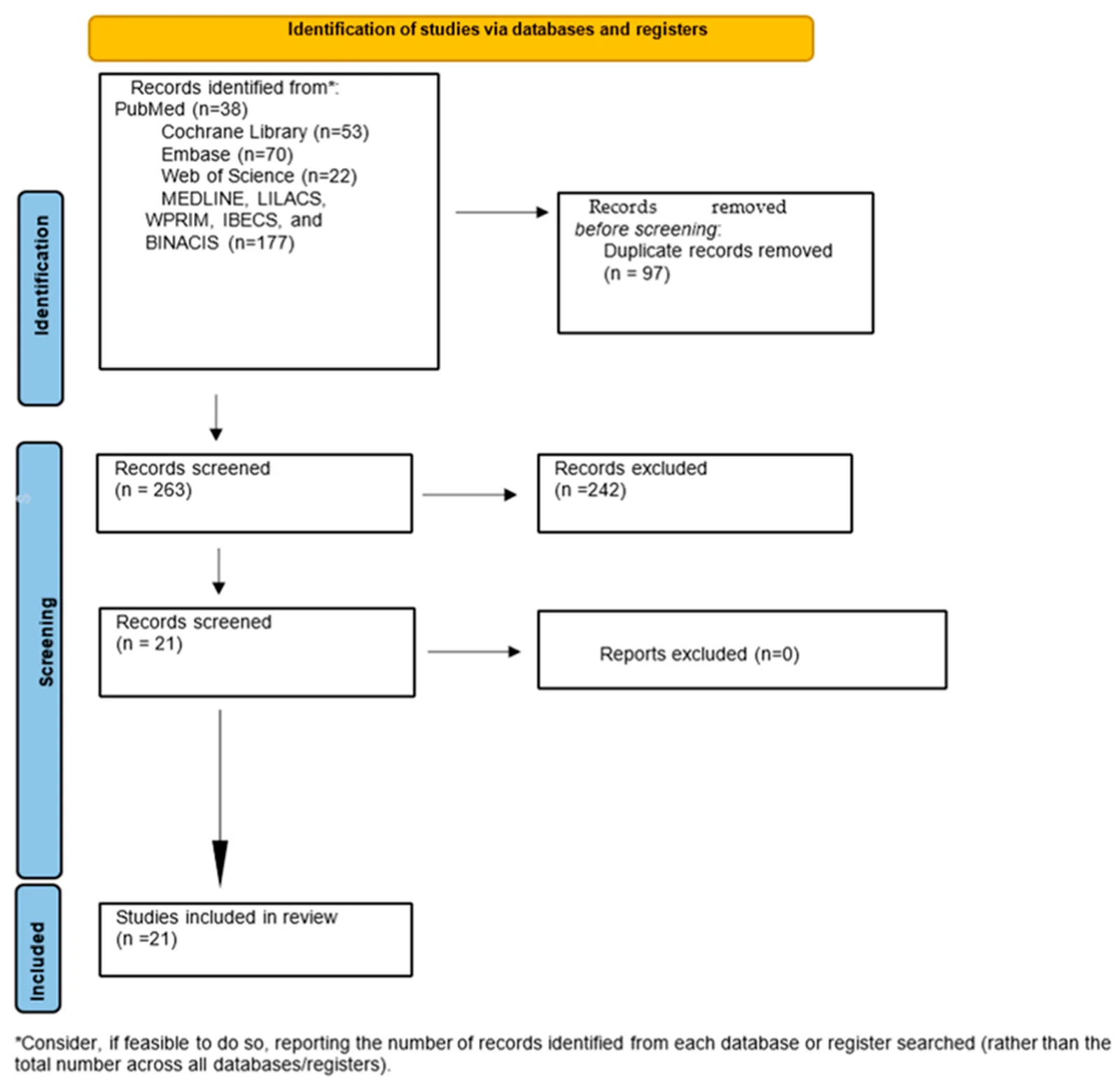
Adapted PRISMA 2020 flow diagram of the identification and recording of studies found in the consulted databases.

All identified records were exported to Rayyan, a tool used for organization, duplicate identification, and study screening [15]. After removing duplicates, study selection was carried out in two stages: screening of titles and abstracts, followed by full-text review. Both stages were performed by two independent reviewers, with blinding enabled in Rayyan to prevent access to the other reviewer’s screening decisions, and a third reviewer resolving any disagreements. No additional automation software was used beyond Rayyan. This scoping review is reported in accordance with PRISMA-ScR [14]. The selection process is presented using an adapted PRISMA 2020 flow diagram [16] (Figure 1) [14,16].

The extraction process was independently conducted by two reviewers using a standardized Excel form, collecting information on the reference, country of study, type of dental procedure analyzed, functional unit, system boundary, life cycle inventory, software, inventory database, environmental impact assessment method, evaluated impact categories, main results, environmental hotspots, and methodological limitations [13]. The extraction form was pilot-tested using five articles to verify the information collected. Disagreements during data extraction and methodological classification were resolved by consensus in a meeting involving a third reviewer. Methodological information was recorded descriptively, distinguishing characteristics explicitly reported in the primary publications from classifications made during the review. This procedure did not constitute a formal assessment of methodological quality or risk of bias.

The results were organized via narrative synthesis and presented through descriptive tables and graphs. Based on a full-text review of the studies, the objects of analysis were grouped into four categories: clinical dental procedures; dental materials and devices; oral health services; and population-level preventive interventions. The categories were assigned during data extraction by the reviewers according to each publication’s primary object of analysis. When a publication addressed more than one analytical area, its primary category was determined by the principal object being assessed, while overlapping features were retained in the descriptive characterization. Furthermore, the methodological characteristics of the selected studies were synthesized, including functional unit, system boundary, software, inventory databases, impact assessment methods, environmental categories analyzed, hotspots, and reported limitations. Environmental hotspots were distinguished according to whether they were explicitly identified by the primary-study authors or inferred during the review’s data extraction and interpretation. The synthesis did not pool environmental estimates or rank alternatives across studies with different functional units, system boundaries, or assessment purposes. Descriptive percentages were calculated using the 21 included publications as the denominator; variables that were not mutually exclusive were not expected to sum to 100%. This organization aimed to describe the scope, distribution, and gaps in the literature on LCA applied to oral health [14].

## 3. Results

The database search resulted in the identification of 360 records, distributed across PubMed (n = 38), Embase (n = 70), Web of Science Core Collection (n = 22), the Cochrane Library (n = 53), and the Virtual Health Library (BVS) interface (n = 177). After export to Rayyan and the removal of 97 duplicates, 263 records remained for screening by title and abstract. Of these, 242 were excluded, as they failed to meet eligibility criteria, and the remaining 21 publications were sought for full-text retrieval. All 21 publications were successfully retrieved and assessed for eligibility, with no exclusions at the full-text stage. Thus, 21 publications were included in the final review. The identification, screening, and inclusion process is presented in an adapted PRISMA 2020 flow diagram (Figure 1).

The included studies were published between 2014 and 2026 [17,18,19,20,21,22,23,24,25,26,27,28,29,30,31,32,33,34,35,36,37], with 20 of the 21 publications (95.2%) dated 2020 or later [18,19,20,21,22,23,24,25,26,27,28,29,30,31,32,33,34,35,36,37]. The distribution by publication year was as follows: 2014 (n = 1) [17], 2020 (n = 2) [18,19], 2021 (n = 2) [20,21], 2022 (n = 5) [24,25,26,27,28], 2023 (n = 3) [22,23,29], 2024 (n = 5) [30,31,32,33,34], 2025 (n = 1) [35], and 2026 (n = 2) [36,37]. The years 2022 and 2024 each accounted for 23.8% (n = 5) [24,25,26,27,28,30,31,32,33,34] of the included publications. Only one publication had a bibliographic publication year before 2020 (4.8%) [17]. The publication years correspond to the final issue year, where available, rather than the date of early-online availability.

The geographical characterization of the included publications was revised to distinguish the settings represented in the assessments from the authors’ institutional affiliations. The available evidence was concentrated in Northern Hemisphere countries [17,18,19,20,21,22,23,24,25,26,27,28,29,30,31,32,33,34,35,36,37], although the geographical context of each assessment requires consideration of the specific setting represented in the original publication. Table 2 presents the characteristics of the included studies, detailing the reference, title, country of conduct, and the object of the LCA analysis. The studies covered various levels of methodological application, including clinical dental procedures (n = 5) [19,26,31,36,37], materials and devices (n = 9) [17,18,21,22,23,24,33,34,35], oral health services(n = 3) [20,30,32], and population-level preventive interventions (n = 4) [25,27,28,29].

The category of clinical dental procedures encompassed studies focused on interventions directly related to individual oral health care. Analyses were identified regarding endodontic treatment [19], personal protective equipment used in dental care during the COVID-19 pandemic [26], nitrous oxide sedation [31], orthognathic surgery [36], and hospital-based tooth extraction [37]. In these studies, the unit of analysis was linked to the procedure performed or the care provided to the patient [19,26,31,36,37].

The category of dental materials and devices included studies on burs [17], toothbrushes [18,34,35], gloves [21], restorative materials [22], interdental hygiene devices [23], clinical examination kits [24], and orthodontic retainers [33]. Generally, these analyses evaluated the stages of production, use, reuse, reprocessing, and disposal of supplies used in dental practice. The social life cycle assessment publication [35] was retained within this analytical category because its primary object was a dental product. However, its social-risk indicators were characterized separately and were not treated as environmental impact outcomes [17,18,21,22,23,24,33,34,35].

The category of oral health services gathered studies that evaluated care delivery and organization beyond a single product or isolated procedure. Analyses were included regarding dental examinations in clinical practice [20], procurement flows, waste, and water consumption in dental services [30], and the overall operation of dental clinics [32]. These studies incorporated various input and output flows, such as energy, materials, water, and waste [20,30,32].

The category of population-level preventive interventions included studies on water fluoridation [25], supervised toothbrushing programs [27,29], and fluoride varnish application [28]. These analyses evaluated promotion and prevention strategies on a collective scale, considering broader timeframes and indirect effects, such as the reduction in future needs for dental treatment [25,27,28,29].

Some studies presented cross-category scopes by combining aspects of materials, clinical processes, and service organization. This was the case for analyses regarding dental care during the COVID-19 pandemic [26] and the procurement of supplies, waste management, and water consumption in dental services [30]. Each publication was assigned to a single primary analytical category according to its main object of assessment, while overlapping characteristics were retained in the descriptive synthesis [26,30].

Regarding system boundaries, cradle-to-grave approaches were documented in assessments of dental burs [17], endodontic treatment [19], and reusable and disposable dental examination kits [24]. Cradle-to-gate approaches were identified in studies of gloves [21], restorative materials [22], and toothbrush materials [34]. Functional units varied according to the analytical object, including individual procedures [19,37], dental products and materials [17,22,24], and population-level preventive interventions [25,27,28,29]. The functional units reported in studies [26] and [30] were also explicitly identified during the methodological data audit. These differences were characterized descriptively rather than interpreted as a direct ranking of methodological quality.

Regarding software, openLCA versions 2 was identified in 17 of the 21 publications (81.0%) [18,19,20,21,23,24,25,26,27,28,29,30,31,32,33,35,37]. SimaPro 9.5 and newer was identified in one publication (4.8%) [34], Umberto (version not reported) in one (4.8%) [22], and the Carebone v5 calculation tool in one (4.8%) [36]. One publication (4.8%) did not identify the software used [17]. In that publication, TRACI 2.0 was identified as an environmental impact assessment method rather than an LCA modelling engine [17]. The ecoinvent inventory database was used in 20 publications (95.2%) [17,18,19,20,21,22,23,24,25,26,27,28,29,30,31,32,33,34,35,37], either alone or in combination with other inventory sources. Its use was recorded at the publication level, without dividing the studies into unsupported exclusive-use and combined-use categories.

Environmental impact assessment methods varied across studies, employing approaches such as TRACI 2.0 [17], ReCiPe [22,34], CML, and ILCD [18,21,24,31,33]. Climate change, expressed in CO_2_ equivalents, was evaluated in the 20 environmental LCA publications (95.2%) [17,18,19,20,21,22,23,24,25,26,27,28,29,30,31,32,33,34,36,37]. Of these, 17 publications (81.0% of the complete review sample) assessed multiple environmental impact categories [17,18,19,20,21,22,23,24,25,26,27,28,31,32,33,34,37], whereas three publications (14.3%) focused on climate change or greenhouse gas emissions [29,30,36]. The dental practice study [32] combined an assessment of 16 environmental impact categories for process-based components with expenditure-based greenhouse gas estimates for procurement. The toothbrush materials study [34] assessed 18 environmental impact categories using ReCiPe Midpoint and examined additional end-of-life carbon scenarios separately. The social-risk indicators assessed in the social LCA publication [35] were reported separately and were not counted as environmental impact categories. Although that publication also reported ancillary greenhouse gas information, its primary analytical framework remained social LCA [35].

The descriptive methodological characterization considered the use of primary and secondary inventory data, the reporting of functional units and system boundaries, modelling assumptions, parameter uncertainty, and sensitivity analyses. The nature and availability of such information varied across the included publications [17,18,19,20,21,22,23,24,25,26,27,28,29,30,31,32,33,34,35,36,37]. An explicitly performed sensitivity analysis was identified in five publications (23.8%) [17,27,28,29,32]. The absence of an explicitly identified sensitivity analysis in the other publications was not automatically interpreted as proof that no uncertainty-related assessment was undertaken. These characteristics were recorded without applying a formal quality score or interpreting differences between methodological approaches as demonstrated deficiencies. Accordingly, unsupported aggregate frequencies of methodological limitations were not retained.

The analysis of environmental contributions identified several recurring areas across the primary publications. These included disposable or single-use materials [19,21,24,26,30], energy consumption [19,20,32,33,37], sterilization and reprocessing [17,24,36,37], transportation [18,25,27,28,32,36,37], and material manufacturing [17,18,21,22,23,33,34]. The reported contributions were extracted at the publication level, while their organization into broader thematic groups was undertaken during the review. This classification did not imply that all primary authors explicitly designated these processes as environmental hotspots or that their relative importance could be compared directly across assessments with different functional units and system boundaries. The social-risk contributors identified in the social LCA publication [35] were not included in this environmental synthesis. Among studies on clinical procedures, the main contributors were related to single-use supplies, energy consumption, and disinfection or sterilization processes, as observed in the analysis of endodontic treatment [19]. In the study of inhalation sedation, nitrous oxide was identified as a particularly relevant contributor to the environmental impact of the assessed procedure [31]. In hospital and surgical procedures, patient transport and energy resource use were also reported as relevant components, particularly in analyses of orthognathic surgery and hospital-based tooth extraction [36,37].

In studies concerning materials and devices, impacts were frequently attributed to industrial production and the supply chain, including raw material extraction, manufacturing, and packaging. This pattern was observed in analyses of restorative materials [22], toothbrushes [18,34], and gloves [21]. In some of these assessments, the use phase made a smaller relative contribution when compared to the initial stages of the life cycle [18,21,22,34]. These findings were interpreted within the boundaries and assumptions of the respective assessments, rather than generalized across different dental products and settings.

Comparisons between alternatives were undertaken in studies of reusable and disposable burs [17], gloves [21], restorative materials [22], dental examination kits [24], and orthodontic retainers [33]. Comparisons between toothbrush designs and materials were also conducted [18,34]. Their findings were described within the functional units and system boundaries adopted by each publication, without pooling environmental estimates across non-equivalent comparisons.

Table 3 presents the descriptive methodological characteristics of the included studies. All percentages use the 21 included publications as the denominator. When variables are not mutually exclusive, the corresponding percentages are not expected to sum to 100%. Publication-level classifications and their supporting references are detailed in Supplementary Table S1.

## 4. Discussion

The application of LCA to oral health is still a nascent field of scientific inquiry. Although the included studies were published between 2014 and 2026, 20 of the 21 publications (95.2%) had bibliographic publication years of 2020 or later [18,19,20,21,22,23,24,25,26,27,28,29,30,31,32,33,34,35,36,37], indicating that the literature identified by this review is predominantly recent. This descriptive distribution should not, however, be interpreted as demonstrating a quantified increase in the overall volume of research over time. This trend aligns with broader literature on sustainability in oral health, which advocates for moving beyond isolated actions and understanding dental practice in terms of its material, operational, and institutional impacts [3,4,5,6]. The specific contribution of this review lies in demonstrating how LCA is beginning to translate this agenda into empirical assessments of products, procedures, services, and preventive interventions [17,18,19,20,21,22,23,24,25,26,27,28,29,30,31,32,33,34,36,37]. The related social LCA publication [35] further illustrates the application of the life cycle framework to a different dimension of sustainability, which was characterized separately from environmental impacts.

The geographic distribution of the studies also defines the scope of the available evidence. The mapped literature was concentrated in Northern Hemisphere countries [17,18,19,20,21,22,23,24,25,26,27,28,29,30,31,32,33,34,35,36,37], although the geographical setting represented in an assessment should be distinguished from the institutional affiliations of its authors. Accordingly, the previously reported percentage attributed to the United Kingdom and Ireland was not retained as a measure of the location of the assessments. This concentration highlights that environmental assessments in healthcare are sensitive to contextual factors, such as energy mixes, supply chains, transportation patterns, healthcare infrastructure, and service delivery models [1,2]. The transferability of numerical LCA estimates may therefore be limited by differences in local electricity generation, procurement practices, transportation distances, waste management systems, sterilization processes, and inventory assumptions. Such contextual variation does not establish that LCA methodology itself is unsuitable for other regions; rather, it emphasizes the need to adapt assessment models to the settings in which they are applied. The development of databases like HealthcareLCA underscores the importance of environmental assessments in healthcare; however, their utility depends on the diversity of the represented contexts [10].

The prevalence of studies on dental materials and devices [17,18,21,22,23,24,33,34,35] demonstrates that supplies play a key role in the application of LCA within oral health. This focus aligns with reviews on sustainability in dentistry, in which the reduction, reuse, recycling, and substitution of materials emerge as recurring strategies to mitigate the environmental impact of oral health services [4,5,6]. However, the studies surveyed add complexity to this discussion: a material’s environmental performance depends not merely on whether it is disposable, reusable, plastic, metallic, or biodegradable, but on how its life cycle is modeled [17,21,22,24,33,34]. Analyses concerning dental burs [17], toothbrushes [18,34], gloves [21], restorative materials [22], interdental hygiene devices [23], clinical kits [24], and orthodontic retainers [33], revealed that the environmental profiles of dental supplies depend on the life cycle stages, processes, and assumptions considered in each assessment. The study addressing toothbrushes from a social impact perspective [35] was analyzed separately and did not contribute social-risk indicators to the synthesis of environmental outcomes. Consequently, the findings do not support a simple hierarchy of “better” or “worse” materials; rather, they indicate that environmental decisions regarding dental supplies require a clear articulation of usage conditions, assumptions regarding reuse and reprocessing, and the specific flows included in the modeling process. In particular, comparisons between reusable and single-use alternatives should be interpreted in relation to product lifespan, frequency of reuse, reprocessing requirements, electricity sources, transportation, and end-of-life assumptions [17,21,24].

Comparisons between studies must be interpreted with caution, as LCA results depend on the choices that structure the model. The included assessments exhibited variation in functional units, system boundaries, inventory sources, impact assessment methods, and modeling assumptions [17,18,19,20,21,22,23,24,25,26,27,28,29,30,31,32,33,34,36,37]. These differences restrict direct comparisons of environmental estimates, even when studies address related dental products or procedures. However, methodological heterogeneity between publications should not automatically be interpreted as evidence of poor methodological quality within individual studies. Likewise, the absence of explicit reporting of a characteristic should be distinguished from evidence that the corresponding methodological procedure was not undertaken. In LCA, differences in the functional unit, system boundaries, inventories, impact assessment methods, and modeling assumptions can alter the magnitude and distribution of results [7,8,9]. The maturity of scientific output in oral health will therefore depend not only on an increase in the number of studies but also on the transparency with which these choices are described and justified. The present review consequently characterized reported methodological features descriptively, without conducting a formal critical appraisal or applying a validated quality-assessment instrument.

Studies focused on clinical dental procedures [19,26,31,36,37] and classified as oral health services [20,30,32] enable an examination of care that goes beyond the specific product used, incorporating flows such as energy, water, equipment, transport, sterilization, reprocessing, waste management, and supply chains. This perspective aligns the findings with the literature on the environmental footprint of health systems, where impacts are understood as the result of supply chains, energy consumption, care infrastructure, and care organization patterns [1,2]. For oral health, the main contribution of these studies is demonstrating that procedures and services cannot be evaluated solely based on what occurs in the dental chair; their environmental burden is also generated by the conditions that make the care possible [19,20,26,30,31,32,36,37]. Nevertheless, the relative contribution of each process depends on the characteristics of the specific clinical or organizational setting, and estimates should not be transferred directly between facilities with different operational conditions.

Population-level preventive interventions [25,27,28,29] introduce a distinct dimension to the environmental discussion in oral health: the future demand for care that can be averted. By evaluating water fluoridation, supervised toothbrushing programs, and fluoride varnish application, these studies shift the focus of LCA toward interventions that address risk, population coverage, and future treatment needs. Where avoided future treatment is included in the assessment model, its estimated environmental contribution depends on assumptions regarding intervention effectiveness, population coverage, time horizon, and the care pathways considered [25,27,28,29]. Such results should therefore not be interpreted as demonstrating identical environmental benefits across all populations or service contexts. This finding resonates with the literature on sustainability in dentistry, which highlights that impact reduction depends not only on individual conduct in the dental office but also on decisions coordinated across clinical practice, prevention, service organization, and institutional responsibility [3,6].

Inhalation sedation with nitrous oxide adds a specific point to the discussion of clinical procedures: in certain interventions, the environmental burden may be concentrated in the therapeutic agent itself, rather than merely in disposable materials or operational care workflows. In the study regarding dental sedation, nitrous oxide played a dominant role in the environmental impact profile, outweighing the relative contribution of the equipment, materials, and processes associated with the care provided [31]. This result is consistent with the literature on sustainable anesthesia. Ryan and Nielsen [38] discussed the global warming potential of inhaled anesthetics in clinical settings, while Sherman et al. [39], in an LCA analysis of anesthetic drugs, demonstrated that emissions associated with inhaled agents can outweigh those from other stages of the life cycle. For dentistry, this evidence challenges a perspective that remains heavily focused on visible waste: in procedures involving substances with high impact potential, the environmental burden of care may lie less in what is discarded at the end of treatment and more in the clinical agent itself that enables the intervention. Nevertheless, this finding pertains to the assessment conditions and environmental indicators considered in the included sedation study [31] and should not be generalized to all dental procedures.

The predominance of the climate change category, expressed in CO_2_-equivalent, demonstrates that LCA in oral health has mirrored the core role carbon emissions play in the environmental debate within the health sector. This indicator was reported in the 20 environmental LCA publications included in this review (95.2%) [17,18,19,20,21,22,23,24,25,26,27,28,29,30,31,32,33,34,36,37], whereas the social-risk indicators in the social LCA publication [35] were considered separately. Nevertheless, the environmental interpretation of an LCA should not be reduced to a single impact category, as different life cycle flows can affect distinct dimensions, such as acidification, eutrophication, human toxicity, resource depletion, and water use [7,8,9]. The inclusion of multiple impact categories in some of the studies indicates that alternatives with lower CO_2_-equivalent emissions do not necessarily perform better across other environmental categories. Importantly, studies reporting climate change as their sole impact category may still employ a life cycle approach; the number of impact categories assessed should not, by itself, determine whether an assessment qualifies as LCA.

Data quality continues to be a key issue for the consolidation of LCA in oral health. The review identified extensive reliance on the ecoinvent inventory database, which was used in 20 of the 21 publications (95.2%) [17,18,19,20,21,22,23,24,25,26,27,28,29,30,31,32,33,34,35,37]. This finding describes a frequently reported inventory source and does not imply that its use was exclusive or that a particular database provides universally superior estimates. The included studies also differed in the availability of primary data, the reporting of parameter uncertainty, and the presentation of sensitivity analyses [17,18,19,20,21,22,23,24,25,26,27,28,29,30,31,32,33,34,36,37]. Because the corresponding classifications were descriptive and not based on a validated quality-assessment instrument, previously reported aggregate frequencies of methodological deficiencies were not retained without study-level verification. These findings do not diminish the utility of LCA, but they do indicate that results must be interpreted in light of the inventory databases used, as well as assumptions regarding service life, transportation, usage load, process efficiency, and final disposal. ISO standards 14040 and 14044 emphasize the need for transparency in defining the goal and scope, conducting the inventory analysis and impact assessment, and interpreting the results [7,8]. In a still-emerging field, the robustness of scientific output will depend less on the proliferation of isolated studies and more on the capacity to generate primary data, test alternative scenarios, and explicitly state the extent to which results are influenced by decisions made during the modeling process.

The identified environmental hotspots demonstrate that the environmental impact of dental practice is not concentrated in a single stage of care. Across the mapped environmental assessments, relevant contributors included disposable or single-use materials [17,21,24,26], energy or electricity consumption [19,20,30,31,32], sterilization or reprocessing [17,19,24], transportation [19,25,30,36,37], and material manufacturing or production [18,21,22,34]. These groupings represent different environmental processes and should not be interpreted as directly comparable categories across heterogeneous assessments. In addition, the review distinguished hotspots explicitly identified by the original study authors from contributions classified during data extraction; the latter are interpretative review-level classifications rather than statements necessarily made by the primary authors. This distribution suggests that oral health sustainability strategies must integrate decisions regarding procurement, the use of disposables, energy efficiency, sterilization protocols, logistics, and supply chain management. Interpreting these hotspots shifts the discussion from a search for isolated solutions toward a more operational analysis of the specific points where dental care generates the greatest environmental burden.

The presence of comparative studies indicates an application of LCA to the evaluation of alternative products and approaches [17,21,22,23,24,33], although the review does not establish that such comparisons are methodologically equivalent or directly transferable to clinical decisions. This result is significant because the practical utility of LCA tends to increase when the analysis allows for the comparison of actual alternatives regarding care, procurement, prevention, or service organization. However, comparisons require equivalent functions, compatible system boundaries, transparent assumptions, and consideration of uncertainty before conclusions about environmental performance can be transferred to decision-making. At the same time, non-comparative studies also play an important role in an emerging field, as they describe inventories, identify sources of impact, and help build an initial evidence base regarding environmental aspects of oral health [19,20,30,31,32,36,37].

This review has certain limitations. First, the heterogeneity of the evaluated objects, functional units, system boundaries, impact assessment methods, and environmental categories limited direct comparisons between the included studies. Second, as this was a scoping review, the synthesis was descriptive in nature and did not aim to estimate aggregate effects, rank alternatives, or define environmentally superior dental practices. Furthermore, no formal critical appraisal of the studies’ methodological quality was conducted, as this is not the primary objective of this type of review. Instead, a descriptive methodological characterization of the included assessments was performed, using methodological dimensions described in ISO 14040 and ISO 14044 as reference points without applying a formal scoring system or validated appraisal tool [7,8]. Finally, data extraction relied on the transparency of reporting in the original studies; therefore, information not explicitly described in the articles may have been classified as absent, partial, or unreported. These classifications should not be interpreted as proof that an unreported procedure was not performed. Additional limitations concern the geographical concentration of the mapped evidence, the dependence of environmental estimates on local modelling assumptions, and the inability of this review to establish decision thresholds or evaluate the certainty of comparative environmental outcomes.

The synthesis of findings indicates that LCA is being incorporated into oral health scientific literature, albeit still concentrated within a limited range of contexts, objects, and methodological traditions [17,18,19,20,21,22,23,24,25,26,27,28,29,30,31,32,33,34,36,37]. The mapped literature shows applications across materials and devices [17,18,21,22,23,24,33,34], clinical procedures [19,26,31,36,37], oral health services [20,30,32], and population-level preventive interventions [25,27,28,29], alongside one related social LCA application [35]. The consolidation of the field will depend on expanding studies across different care settings, generating primary data, explicitly stating modeling premises, and employing sensitivity analyses more consistently. This approach aligns with the rigor expected in LCA studies, where the strength of conclusions relies on the coherence among the objective, scope, inventory, impact assessment, and interpretation of results [7,8]. As a scoping review, this study does not define environmentally superior dental practices; rather, it maps where LCA has been applied and identifies the gaps that still limit its consolidation as a decision-support tool in oral health. The findings therefore provide a descriptive basis for future research, while clinical, institutional, and policy recommendations require context-specific assessments, functionally equivalent comparisons, and explicit consideration of uncertainty.

## 5. Conclusions

This scoping review mapped 21 publications addressing life cycle approaches in oral healthcare, including environmental LCA applications involving dental materials and devices, clinical procedures, care services, and population-level preventive interventions. Findings indicate that the environmental impacts of dental care involve different contributing processes, including the use of disposable materials, energy consumption, sterilization and reprocessing processes, transportation, and material manufacturing. The relative importance of these contributors depends on the assessment context, system boundaries, and modelling assumptions, and the mapped evidence does not establish a universal ranking of environmental hotspots. The diversity of objects evaluated, functional units, system boundaries, data sources, impact categories, and the reporting of sensitivity analyses limit direct comparison between studies. Furthermore, the concentration of research output in Northern Hemisphere countries, alongside the geographical and methodological characteristics of the included publications [17,18,19,20,21,22,23,24,25,26,27,28,29,30,31,32,33,34,35,36,37], highlights the need to expand investigations across different care settings and geographic regions. Rather than identifying environmentally superior dental practices or establishing direct clinical, institutional, and policy recommendations, this scoping review provides a descriptive mapping of methodological characteristics, environmental contributors, and research gaps. Future decision-oriented applications of LCA require context-specific assessments, functionally equivalent comparisons, transparent modelling assumptions, the generation of primary data where feasible, and explicit consideration of uncertainty.

## Figures and Tables

**Table 1:** Detailed breakdown of the PCC structure used to guide the scoping review.

PCC Element	Definition Adopted in the Review	Operationalization
P—Population	Procedures, products, materials, technologies, and services related to oral health	Studies involving clinical dental procedures, dental materials and devices, oral health services, or population-level preventive interventions.
C—Concept	LCA applied to the measurement of environmental impacts	Studies that used LCA to assess environmental impacts, considering elements such as functional unit, system boundary, life cycle inventory, software, database, impact assessment method, environmental categories, and hotspots.
C—Context	Dental and oral health settings at different levels of care or organization	Dental clinics, hospitals, dental laboratories, oral health services, community settings, and population-based interventions related to oral health promotion, prevention, diagnosis, or treatment.

**Table 2:** Characteristics of the 21 publications included in the scoping review, according to author, bibliographic publication year, geographical assessment context, object of life cycle assessment, and primary analytical category.

Ref.	Author, Year	Country/Location	Object of LCA Analysis	Analytical Category
[17]	Unger; Landis, 2014	United States	Reusable versus disposable dental burs	Dental materials and devices
[18]	Lyne et al., 2020	United Kingdom	Toothbrush as a model for integrating evidence-based health and environmental sustainability	Dental materials and devices
[19]	Duane et al., 2020	Sweden	Endodontic treatment	Clinical dental procedures
[20]	Borglin et al., 2021	Sweden	Dental examination in a hypothetical clinical practice	Oral health services
[21]	Jamal et al., 2021	United Kingdom	Non-sterile examination gloves and sterile surgical gloves	Dental materials and devices
[22]	Smith et al., 2022	United Kingdom	Dental restorative materials	Dental materials and devices
[23]	Abed et al., 2022	United Kingdom	Interdental hygiene aids	Dental materials and devices
[24]	Byrne et al., 2022	Ireland	Reusable and disposable dental examination kits	Dental materials and devices
[25]	Duane et al., 2022	Ireland	Water fluoridation used as community-based caries prevention intervention	Population-level preventive interventions
[26]	Almutairi et al., 2022	Ireland	Dental care in the context of the COVID-19 pandemic	Clinical dental procedures
[27]	Ashley et al., 2022	United Kingdom	Tooth-brushing programs used as community-based intervention for caries prevention	Population-level preventive interventions
[28]	Lyne et al., 2022	United Kingdom	Application of fluoride varnish as a community-based caries prevention intervention	Population-level preventive interventions
[29]	Bakar et al., 2023	Scotland	Supervised toothbrushing program for children	Population-level preventive interventions
[30]	Suresh et al., 2024	United Kingdom	Acquisition of supplies, waste generation, and water consumption in dental practice	Oral health services
[31]	Fennell-Wells et al., 2024	United Kingdom	Inhalation sedation with nitrous oxide and equipment used in dentistry	Clinical dental procedures
[32]	Duane; Steinbach, 2024	United Kingdom	Environmental footprint of a dental clinic	Oral health services
[33]	Tan et al., 2024	Ireland	Post-treatment orthodontic retainers: Hawley and Essix	Dental materials and devices
[34]	Mazur et al., 2024	Poland	Materials constituting manual toothbrushes	Dental materials and devices
[35]	Duane et al., 2025	Ireland	Social life cycle assessment of toothbrushes and associated social-risk indicators	Dental materials and devices
[36]	Mattei et al., 2026	France	Orthognathic surgery	Clinical dental procedures
[37]	Künzle et al., 2026	Germany	Tooth extraction in a university hospital setting	Clinical dental procedures

Note: Publication years correspond to the final journal issue year, where available. Each publication was assigned to one primary analytical category according to its main object of assessment. Publications addressing overlapping analytical areas were categorized by their primary object, while secondary characteristics were retained in the descriptive synthesis. Geographical assignments reflect the settings represented in the assessments rather than author affiliations alone. The social life cycle assessment publication [35] was identified separately from environmental LCA studies.

**Table 3:** Quantitative summary of verified methodological and analytical characteristics of the 21 publications included in the scoping review.

Dimension	Predominant or Relevant Category	n	%	Studies Included in the Numerator
Assessment type	Environmental life cycle assessment	20	95.2%	[17,18,19,20,21,22,23,24,25,26,27,28,29,30,31,32,33,34,36,37]
Assessment type	Social life cycle assessment	1	4.8%	[35]
Object of analysis	Clinical dental procedures	5	23.8%	[19,26,31,36,37]
Object of analysis	Dental materials and devices	9	42.9%	[17,18,21,22,23,24,33,34,35]
Object of analysis	Oral health services	3	14.3%	[20,30,32]
Object of analysis	Population-leve preventive interventions	4	19%	[25,27,28,29]
Software	OpenLCA	17	81%	[18,19,20,21,23,24,25,26,27,28,29,30,31,32,33,35,37]
Software	SimaPro	1	4.8%	[34]
Software	Umberto	1	4.8%	[22]
Software	Carebone calculation tool	1	4.8%	[36]
Software	Not specified	1	4.8%	[17]
Inventory base	Ecoinvent, isolated or combined with other sources	19	90.4%	[17,18,19,20,21,22,23,24,25,26,27,28,29,30,31,32,33,34,35]
Inventory base	Other banks or sources	1	4.8%	[36]
Inventory base	Not specified	1	4.8%	[37]
Impact categories	Multiple environmental impact categories	17	81%	[17,18,19,20,21,22,23,24,25,26,27,28,31,32,33,34,37]
Impact categories	Climate-change-focused environmental assessment	3	14.3%	[29,30,36]
Impact categories	Social LCA with ancillary greenhouse gas reporting	1	4.8%	[35]
Methodological reporting	Identifiable functional or reference unit	21	100%	[17,18,19,20,21,22,23,24,25,26,27,28,29,30,31,32,33,34,35,36,37]
Methodological reporting	Explicit sensitivity analysis performed	5	23.8%	[17,27,28,29,32]

Note: Absolute frequencies (n) and percentages were calculated using the 21 included publications as the denominator. The social life cycle assessment publication [35] was identified separately from environmental LCA studies, and its social-risk indicators were not classified as environmental impact outcomes. Software categories describe the modelling engines or calculation tools identified in the publications; software used only for auxiliary purposes was not classified as an LCA modelling engine. Any use of ecoinvent was recorded regardless of whether additional inventory sources were employed. The table describes reported methodological characteristics and does not constitute a formal quality assessment. An identifiable functional or reference unit does not constitute evidence of methodological quality. No formal critical appraisal or methodological quality scoring was conducted.

## Data Availability

The raw data supporting the conclusions of this article will be made available by the authors on request.

## Supplementary Materials

The following supporting information can be downloaded at: https://www.mdpi.com/article/10.3390/healthcare14213600/s1, Table S1: Traceability matrix of methodological and analytical characteristics of the studies included in the scoping review.

## Abbreviations

The following abbreviations are used in this manuscript:

LCA	Life Cycle Assessment
PRISMA-ScR	Preferred Reporting Items for Systematic Reviews and Meta-Analyses Extension for Scoping Reviews
OSF	Open Science Framework
PCC	Population, Concept, and Context

## Appendix A. Search Strategy Used in Scoping Review

**Table A1:** Search strategy in electronic databases.

Electronic Database	Search Strategy
PubMed	“Dentistry”[MeSH Terms] OR “Dental Equipment”[MeSH Terms] OR “equipment dental”[All Fields] OR “Dental Equipments”[All Fields] OR “Dental Instruments”[MeSH Terms] OR “instrument dental”[All Fields] OR “Dental Instrument”[All Fields] OR “instruments dental”[All Fields] OR “Dental Health Services”[MeSH Terms] OR “health services dental”[All Fields] OR “Dental Health Service”[All Fields] OR “health service dental”[All Fields] OR “service dental health”[All Fields] OR “oral healthcare”[All Fields] OR “oral hygiene aid”[All Fields]) AND (“life cycle assessment”[All Fields] OR “life cycle analysis”[All Fields] OR “life cycle impact assessment”[All Fields] OR “LCA”[All Fields] OR “cradle to grave”[All Fields]
Embase	‘Dentistry’/exp OR ‘Dentistry’/syn ‘Dental Equipment’/exp OR ‘Dental Equipment’/syn ‘Dental Instruments’/exp OR ‘Dental Instruments’/syn ‘Dental Health Services’/exp OR ‘Dental Health Services’/exp ‘oral healthcare’/exp ‘oral hygiene aid’/syn) AND (‘life cycle assessment‘/exp OR ‘life cycle assessment‘/syn ‘life cycle analysis’/exp OR ‘life cycle analysis’/syn ‘life cycle impact assessment’/exp OR ‘life cycle impact assessment’/syn ‘cradle to grave’/exp OR ‘cradle to grave’/syn
Web of Science and Scopus	TW: (Dentistry) OR (Dental Equipment) OR (Dental Instruments) OR (‘Dental Health Services) OR (oral healthcare) OR (oral hygiene aid) and (life cycle assessment) OR (life cycle analysis) OR (life cycle impact assessment) OR (LCA) OR (cradle to grave)
LILACS	“Odontologia” OR “Dentistry” OR “Odontología” OR “Odontologie” OR “Equipamentos Odontológicos” OR “Dental Equipment” OR “Equipo Dental” OR “Équipement dentaire” OR “Instrumentos Odontológicos” OR “Dental Instruments” OR “Instrumentos Dentales” OR “Instruments dentaires” OR “Serviços de Saúde Bucal” OR “Dental Health Services” OR “Servicios de Salud Dental” OR “Services de santé buccodentaire” OR “oral healthcare” OR “oral hygiene aid”
WPRIM	“Odontologia” OR “Dentistry” OR “Odontología” OR “Odontologie” OR “Equipamentos Odontológicos” OR “Dental Equipment” OR “Equipo Dental” OR “Équipement dentaire” OR “Instrumentos Odontológicos” OR “Dental Instruments” OR “Instrumentos Dentales” OR “Instruments dentaires” OR “Serviços de Saúde Bucal” OR “Dental Health Services” OR “Servicios de Salud Dental” OR “Services de santé buccodentaire” OR “oral healthcare” OR “oral hygiene aid”
IBECS	“Odontologia” OR “Dentistry” OR “Odontología” OR “Odontologie” OR “Equipamentos Odontológicos” OR “Dental Equipment” OR “Equipo Dental” OR “Équipement dentaire” OR “Instrumentos Odontológicos” OR “Dental Instruments” OR “Instrumentos Dentales” OR “Instruments dentaires” OR “Serviços de Saúde Bucal” OR “Dental Health Services” OR “Servicios de Salud Dental” OR “Services de santé buccodentaire” OR “oral healthcare” OR “oral hygiene aid”
BINACIS	“Odontologia” OR “Dentistry” OR “Odontología” OR “Odontologie” OR “Equipamentos Odontológicos” OR “Dental Equipment” OR “Equipo Dental” OR “Équipement dentaire” OR “Instrumentos Odontológicos” OR “Dental Instruments” OR “Instrumentos Dentales” OR “Instruments dentaires” OR “Serviços de Saúde Bucal” OR “Dental Health Services” OR “Servicios de Salud Dental” OR “Services de santé buccodentaire” OR “oral healthcare” OR “oral hygiene aid”
Cochrane Library	#1 MeSH descriptor: [Dentistry] explode all trees #2 MeSH descriptor: [Dental Equipment] explode all trees #3 MeSH descriptor: [Dental Instruments] explode all trees #4 MeSH descriptor: [Dental Health Services] explode all trees #5 oral healthcare #6 oral hygiene aid #7 #1 OR #2 OR #3 OR #4 OR #5 OR #6 #8 life cycle assessment #9 life cycle analysis #10 life cycle impact assessment #11 LCA #12 cradle to grave #13 #8 OR #9 OR #10 OR #11 OR #12 #14 #7 AND #13
