# Supplementary material for: Life Cycle Assessment Applied to Oral Healthcare: A Scoping Review
Source: Healthcare (Basel). 2026 Oct 23;14(21):3600. doi: 10.3390/healthcare14213600 (PMC13763498; doi:10.3390/healthcare14213600)
Supplement: Supplementary file 1 [file healthcare-14-03600-s001.zip › healthcare-4613319-supplementary.pdf]

Supplementary Table S1. Publication-level traceability matrix of methodological and analytical characteristics of the 21 publications included in the scoping review.

Ref.	Author, year	Functional unit or assessment unit	System boundary	Software and inventory sources	Impact assessment method / coverage	Main contributors reported
[17]	Unger; Landis, 2014	One reusable dental bur used up to 30 times, compared with 30 disposable burs	Cradle-to-grave	Modelling engine not identified; ecoinvent v2.2, USLCI, ELCD	TRACI 2.0; multiple environmental categories	Packaging, autoclaving, air freight and ultrasonic cleaning, depending on the scenario
[18]	Lyne et al., 2020	Toothbrush use by one person over five years	Cradle-to-grave	openLCA; ecoinvent v3.5	PEF, IPCC GWP100, ILCD, CML-IA, AWARE; multiple categories	Toothbrush materials, transportation and consumer water use
[19]	Duane et al., 2020	One complete root canal treatment involving two appointments	Cradle-to-grave	openLCA; ecoinvent v3.5	Multiple environmental categories; specific LCIA method not fully identified	Electricity, endodontic instruments, cleaning products and disposable materials

[20]	Borglin et al., 2021	One dental examination per patient	Cradle-to-grave	openLCA; ecoinvent v3.5	PEF, ILCD, CML, IPCC GWP100, AWARE	Detergents, disposable bibs, disinfectants, instruments and water
[21]	Jamal et al., 2021	One pair of gloves used by a healthcare professional	Cradle-to-grave	openLCA; ecoinvent v3.7.1	ILCD, CML, IPCC GWP100, AWARE, ReCiPe	Glove manufacturing and nitrile raw materials
[22]	Smith et al., 2023	One direct dental restoration per restorative material system	Cradle-to-gate	Umberto LCA+; ecoinvent	ReCiPe Midpoint v1.13; multiple categories	Silver in amalgam, material-processing energy and packaging
[23]	Abed et al., 2023	Daily interdental cleaning by one person over five years	Cradle-to-grave	openLCA; ecoinvent v3.7	ILCD, IPCC GWP100, CML, ReCiPe	Polypropylene floss-pick handles, raw material production and packaging

[24]	Byrne et al., 2022	One dental examination kit used for one patient	Cradle-to-grave	openLCA; ecoinvent v3.7.1	PEF, ReCiPe Endpoint; multiple categories	Disposable-kit manufacturing and reusable-kit sterilization
[25]	Duane et al., 2022	Water fluoridation for one five-year-old child over one year	Cradle-to-grave	openLCA; ecoinvent v3.7.1	ILCD, IPCC GWP100, CML, AWARE, ReCiPe	Reagent transportation, sulfuric acid production and chemical processing
[26]	Almutairi et al., 2022	PPE required for one patient during one dental appointment	Cradle-to-grave	openLCA; ecoinvent v3.7.1	ReCiPe 2016 Endpoint; PEF normalization; multiple categories	PPE manufacture, raw materials, packaging, water consumption and incineration
[27]	Ashley et al., 2022	One child receiving a toothbrushing prevention programme for one year	Cradle-to-grave	openLCA; ecoinvent v3.7.1	ILCD, IPCC GWP100, CML, AWARE, ReCiPe	Staff travel, tap water, toothbrushes and toothpaste

[28]	Lyne et al., 2022	Two fluoride varnish applications for one child during one year	Cradle-to-grave	openLCA; ecoinvent v3.7.1	ILCD, IPCC GWP100, CML, AWARE, ReCiPe	Patient/staff travel, examination kits and fluoride varnish
[29]	Bakar et al., 2023	Annual supervised toothbrushing per child and modelled avoided dental treatments	Cradle-to-grave	openLCA; ecoinvent v3.7.1	Climate change (CO_2e), using PEF guidance	Emissions associated with modelled avoided treatments, particularly general anaesthesia and transportation
[30]	Suresh et al., 2024	Seven functional units covering correspondence, supplies, water use and waste	Cradle-to-grave	openLCA; ecoinvent v3.8	Climate change (gCO_2e); PEF guidance	Incineration, mains water, disposable syringe tips and dishwashing
[31]	Fennell-Wells et al., 2024	Thirty minutes of inhalation sedation with 50:50 N_2O/O_2 at 6 L/min	Cradle-to-grave	openLCA; ecoinvent v3.8	ILCD, IPCC GWP100, CML, AWARE; multiple categories	Direct N_2O emissions, equipment production and disposal

[32]	Duane; Steinbach, 2024	One full-time dental practice operating 220 days per year	Hybrid process-based and spend-based modelling	openLCA; ecoinvent v3.9.1, DEFRA factors	Sixteen environmental impact categories for physical processes; expenditure-based GHG estimates for procurement	Patient/staff travel, procurement, natural gas and electricity
[33]	Tan et al., 2024	Production of one post-orthodontic retainer	Cradle-to-grave, with common comparative stages excluded	openLCA; ecoinvent v3.7.1	PEF, IPCC GWP100, ILCD, CML, ReCiPe	Manufacturing materials and laboratory energy
[34]	Mazur et al., 2024	Production of one manual toothbrush; separate carbon disposal scenarios	Mixed/discordant boundaries: production-focused LCIA and separate end-of-life scenarios	SimaPro 9.5; ecoinvent v3.8	ReCiPe Midpoint (I), 18 categories; additional disposal carbon scenarios	Toothbrush mass, bristle and handle composition, and modelled disposal scenarios
[35]	Duane et al., 2025	Twenty toothbrushes used over five years	Cradle-to-grave social LCA	openLCA; ecoinvent v3.9, Soca v2 / PSILCA	Social-risk indicators in medium-risk hours; ancillary GHG reporting	Social risks associated with supply chains, labour conditions, wages and governance

[36]	Mattei et al., 2026	One complete orthognathic surgery patient pathway through postoperative follow-up	Patient-pathway carbon assessment using GHG Protocol Scopes 1–3	Carebone; internal calculation database	GWP100; climate change	Patient transportation, medical devices, sterilization and anaesthetic gases
[37]	Künzle et al., 2026	One complete tooth extraction appointment, including consent procedures	Cradle-to-grave	openLCA; ecoinvent v3.9.1	PEF harmonisation approach; 16 environmental categories	Patient/staff travel, sterilization steam, electricity and cleaning products

Notes: LCA, life cycle assessment; LCIA, life cycle impact assessment; PEF, Product Environmental Footprint; PPE, personal protective equipment; GHG, greenhouse gases.

System boundaries were characterized using both the descriptions provided by the primary-study authors and the life cycle stages effectively represented in their models. Publications employing different environmental assessment approaches were not automatically considered methodologically equivalent.

The environmental assessment in [32] combined process-based modelling covering 16 environmental impact categories with expenditure-based greenhouse gas estimates for procurement. In [34], the primary life cycle impact assessment focused on toothbrush production, while end-of-life carbon scenarios were modelled separately.

The social LCA publication [35] was retained as a related life cycle application but was not included in the environmental impact categories or environmental hotspot synthesis.

Explicit sensitivity analyses were identified in five publications [17,27–29,32]. For the remaining publications, the absence of an explicitly identified sensitivity analysis was not automatically interpreted as proof that no uncertainty-related assessment was undertaken.

The main contributors presented in this table summarize findings reported in the original publications. Their grouping into broader environmental themes was undertaken during the review and should not be interpreted as a universal ranking of environmental hotspots.
